# Development of a dual point humidity sensor using POF based on twisted fiber structure

**DOI:** 10.1038/s41598-024-59853-3

**Published:** 2024-05-10

**Authors:** Sadam Hussian, Mujahid Mehdi, Abdul Ghaffar, Kun Lan, Yanjun Hu, Huan Lin, Mumtaz A. Qaisrani, Sikandar Ali, Jie Lin, Rehan Mehdi, Rui Ma

**Affiliations:** 1 Key Laboratory of Air-Driven Equipment Technology of Zhejiang Province, College of Mechanical Engineering, Quzhou University, Quzhou, 32400 Zhejiang China; 2 Faculty of Design, Aror University of Art Architecture Design & Heritage Sindh, Sukkur, 65200 Pakistan; 3 Taiyuan Institute of Technology, Taiyuan, China; 4 School of Mechanical & Materials Engineering, University College Dublin, Dublin, Ireland

**Keywords:** Dual point sensor, Fiber twisting, Humidity sensor, Light side coupling, Polymer optical fiber, Electrical and electronic engineering, Optical techniques, Techniques and instrumentation

## Abstract

The humidity has often been measured through a single point sensor. Where, the humidity could be varied at different locations as well as depending on environmental conditions. The present paper developed the dual point humidity measuring sensor by using a polymer optical fiber (POF) based on a single illuminating fiber. The sensor’s basic structure is to twist two fibers and bend them at a certain radius. However, the dual point sensor is developed through the cascading of twisted micro bend (TMB-1 and TMB-2). The twisting of fibers couples the light from one fiber to another fiber through the side coupling method. An increase in the humidity level leads to a change in the reflective index, which helps to get variation in coupled light intensity. To measure the humidity, the dual point sensors are placed into the control humidity chamber at two random positions. The power reading variation is significantly linear when the humidity level increases from 30 to 80%. The sensor has a fast response of about 1 s and a recovery time of about 4 s. Furthermore, the chemical coating is applied to improve the sensor’s sensitivity. Between 30 and 80% range of humidity, the both sensors of dual point TMB-1 and TMB-2 have appropriate sensitivity and detection limits, which is about 680.8 nW/% and 763.9 nW/% and 1.37% and 1.98%, respectively. To measure the humidity at variable positions, the present dual points humidity sensor is well-stable, easy, and straightforward, which uses a less expensive method.

## Introduction

Humidity is an important factor of ambient air quality. The increasing humidity in the atmosphere impacts environmental air quality, which produces numerous environmental challenges. The challenges for human beings include health problems from respiratory to sick diseases^1–4^. Humidity control is required in living rooms, hospitals, production industries, warehouse goods, cultural and historical buildings, and many more places^5–8^. Before moving on to controlling the humidity, the humidity measurement is the basic and necessary step. Several techniques are being applied for measuring humidity, such as hygrometer, psychrometer, dew point meter, humidity data logger etc^9–12^. It’s worth noting that the accuracy and reliability of the humidity measurement of the above techniques always vary due to the quality and measuring method of the instrument used. The calibration and maintenance of instruments are required to get the most appropriate results. The calibration and maintenance of instruments make it complex and expensive. Besides the above techniques of humidity measurement, the polymer optical fiber humidity sensor is also widely applied to measure humidity^13^. The optical fiber sensor has certain advantages over electronic counterpart sensors, such as safety, fast response, cost effective, simple structure etc^14–17^.

Up to present, several researchers have been demonstrating different types of polymer optical fiber sensors based on the fiber material and configuration. The sensors include the macroband fiber^18^, tapered fiber^19^, fiber brag gratings^20^. These sensors work on the principle of passing light from the fiber, where the light power variation is detected by the change in the reflective index R.I. The R.I changed due to the humidity intensity variation. Further, the variation in the light power measurement highlights the humidity value. Rao et al.^21^ reviewed the optical humidity sensors. Their study compiled the number of different types of humidity sensors based on the optical method. Ascorbe et al.^22^ combined the study of the recent development of fiber optical based humidity sensors. Their combined study indicates that the optical fiber has proposed numerous sensors that have significant advantages over the electronic base sensors. Further, their study suggested recommendations for future improvement in the optical fibers. Peng et al.^23^ proposed the U-shaped twisted microfiber to sense the humidity. Their proposed sensor is capable to sense the humidity in the range of 18% to 95% with a sensitivity of 100.2 pm/%RH.

Fiber Bragg Grating (FBG) is a step toward measuring the humidity at different positions for the entire sensing fiber length. Li et al.^24^ reviewed the FBG for quasi-distributed application. The FBG is successfully applied for different applications to sense the respective parameters at different positions. Theodosiou et al.^25^ fitted the FBG based humidity sensor in the concrete slab. Their designed sensor is capable of sensing the humidity in the concrete slab. Zhang et al.^26^ investigated the response time of the FBG humidity sensor. For improving sensor’s response time, the FBG humidity sensor were treated with acetone chemically. In the result of etching acetone chemical, the sensor’s response is increased. Which leads in fast response, as for humidity level increasing and decreasing conditions, the sensor could response within seven and twelve minutes. The FBG is not very sensitive to measuring humidity. The coating could be applied to increase the sensitivity of FBGs and make it capable to the humidity measuring Dong et al.^27^. Measured the humidity through FBG humidity sensor. To prepare the FBG humidity sensor, the FBGs were coated with Polyvinyl alcohol. Their proposed sensor can measure the humidity from 30 to 95% with a sensitivity of 0.737 nW/%. Correia et al.^28^ increased the sensitivity and durability by applying hybrid material coatings on the FBGs. By the applying hybrid material coatings, the sensitivity of the FBG sensor is significantly improved.

The present work proposes the cost effective and inherent safe dual point sensor to measure the humidity for distinct positions based on the single light source. Our proposed dual point humidity sensors offer several advantages, including the ability to monitor humidity at two variable positions by using a single control system. This can be particularly useful in applications requiring distributed or localized humidity measurements, such as environmental monitoring, industrial processes, building automation systems, etc. To design dual point sensor for proposed study, two fibers are twisted and bent. The twisting and bending of fibers are performed two times as proposed dual point sensor could be designed. For measuring humidity, the designed dual point sensor is placed in the humidity chamber at two variable locations. In dual point sensor, the illuminating input fiber is common, which is joined with power source LED. Where to measure humidity, the twisted and bended two fiber’s output ends are connected with separate power meters. The present paper proposed dual point sensors have effectively measured the humidity in the range of 30% to 80% with liner response in humidity ascending and descending values. Both sensors (TMB-1 and TMB-2) of dual point sensor have good sensitivity (680.8 nW/% and 763.9 nW/%) with detection limits of (1.37% and 1.98%), respectively. Further, the dual point sensors are timely response with respect to variation in the humidity level within 1 s. The contents of the present study are subdivided in different sections. The first section has reviewed literature about the effect of humidity and measuring methods. The second section presents information about experimental setup and sensor construction. Further, in the third section, the findings of the proposed dual point sensor of the present work are discussed. The last section of the conclusions highlights the main findings about present proposed dual point sensor.

## Experimental setup

### Fiber material and measuring instruments

The present work proposes a dual point sensor to measure the humidity variation at different points based on the single light illuminating fiber. For sensor fabrication; the 3 m polymethyl methacrylate (PMMA) fiber is used. The SK-40 fiber was purchased from Mitsubishi company. The fiber has a core diameter of 980 µm and cladding thickness of 10 µm. Further, the fiber has core and cladding reflective indexes of 1.492 and 1.402, respectively. The PMMA long fiber was cut into pieces for designing the dual point sensor structure. To illuminate the PMMA fiber, the long piece of primary fiber is attached to the LED light source. For illuminating primary fiber, the LED light source of Thorlabs 660 nm wavelength was used. The light intensity of the LED is controlled with the Thorlabs (LEDD1B) driver unit. Further, to measure the light at output, the sensor fibers forward ends are attached to the power meters of Thorlabs (PM100USB). The secondary fibers gain light from the primary fiber which is attached to the LED power source based on the side couplings principle. For side couplings, the secondary fibers were tightly twisted with the primary fiber at two different positions, respectively.

### Sensor's fabrication

**Figure 1:**
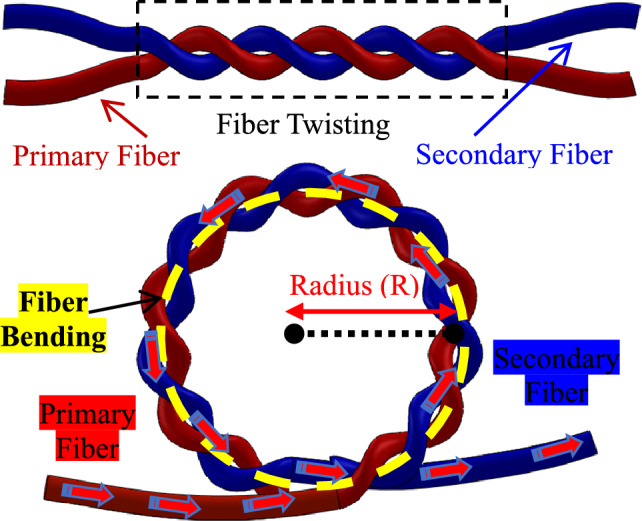
Sensor basic structure.

The proposed dual point humidity sensor of the present study is fabricated from the PMMA fiber. The sensor design is based on the two fibers twisted with each other. After twisting of fibers, the twisted fibers were macro-bent, the structure named as the Twisted Micro Bend (i.e. TMB-1 and TMB-2). The twisting of two pieces of fibers results in the gap between two fibers. In the twisted gap, the R.I could be one because of the air gap. Once the basic structure of dual point sensor is achieved by twisting two fibers, the twisted fibers were bent as more light could be radiated from the illuminating fiber. The dual point humidity sensor TMB-1 and TMB-2 are controlled through a single-input signal system that uses a single-light-input LED control unit, which is connected with primary fiber having aim to illuminate fiber. While, for sensing the humidity variation, both sensors TMB-1 and TMB-2 have produced the output signal independently by connecting forward end of secondary fibers with two different power meters. The sensor fabrication consists two secondarily fibers independent twisting with the primary fiber at individual positions (see Fig. 1). Figure 1 presents the basic sensor fabrication structure of the two fibers twisting and bending. Further, to minimize the external surrounding light influence, the naked fibers after bend structure were covered with a black tube. After the twisted structure of sensor, there is still a gap between two fibers.

The gap between twisted fibers was chemically treated. The sensor's chemical treatment helps to absorb the settling water vapor of fiber. For chemical treatment, the coating of the agarose-gel is applied. By applying agarose-gel coating, the sensor's sensitivity is significantly increased. Many coating materials are widely used in previous work by a number of researchers. The agarose-gel with 0.5% is applied in the present work, which may not affect as a medium^32^. The solution of the agarose-gel is produced by putting (MS-H280-Pro, Dragonlab, Beijing, China) in distilled water. To make the solution of the agarose-gel, the water is heated up to 65 °C. A 0.5 wt% agarose (A6013, Sigma Aldrich, St. Louis, MO, USA) was dissolved in it. The final product of agarose-gel was applied in the gaps between twisted fiber with the help of the syringe. After agarose-gel coted on the twisted fiber sensor, then let it dry for 24 h at room temperature.

### Sensor integration into humidity chamber

**Figure 2:**
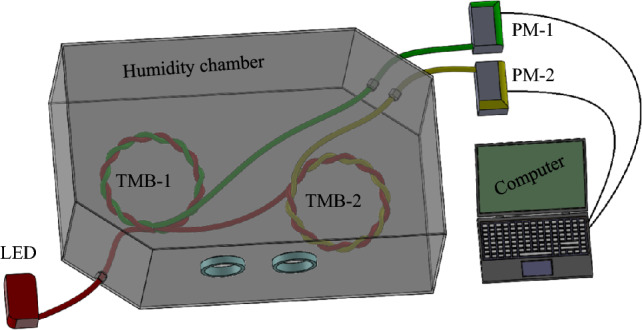
Schematic diagram of experimental setup.

After successfully fabrication of the dual point humidity sensor, the sensors were integrated into the humidity chamber. Figure 2 shows the schematic three-dimensional view of the humidity chamber with integrated sensors TMB-1 and TMB-2. The placement of the both sensors TMB-1 and TMB-2 are used at the bottom surface of the humidity chamber at a certain gap. The light controlling and measuring equipment LED power source and power meters are placed outside the humidity chamber. The humidity chamber only contains the TMB-1 and TMB-2 (i.e., fabricated sensors). Further, the marketable sensor thermos-hygrometer (AH8008 AOSONG, Guangzhou, China) was also fitted into the experimental setup to calibrate the humidity. Based on the reference thermos-hygrometer sensors, the present sensor has been validated the sensing capability. The humidity sensing of the present work with respect to thermos-hygrometer were performed at a room temperature of 25 °C. Further, for input power to illuminate the fibers, 20 mW was applied.

### Sensor's working principle

The basic phenomenon of the sensor is based on the macro-bend loss to sense the humidity. Fibers are twisted and bent together to make the sensor basic structure. Using the side coupling principle, two fibers are twisted and bent to couple light between them. As previously, several researchers have been coupled light through this side coupling principle^29^. Further, the side coupling could be subdivided into two basic branches. Such as twisted macro bend coupling method TMBC^30^ and the cladding mode frustrated total internal reflection CMFTIR^31^. The R.I. has a significant role for light coupling, as air and liquid have different R.I. The increasing humidity level in the air also changes the medium. Thus, the present work senses the humidity at dual point based on the change in R.I. As above Fig. 2 presented the overall experimental setup with the integration of the sensors into the humidity chamber. The experimental setup consists of the TMB-1 and TMB-2, LED light source, power meter, and humidity chamber. Light passes from the primary fiber through a LED light source to illuminate it. The small amounts of light from primary fiber is transferred into the secondary fiber at the twisted sensor structure TMB-1 and TMB-2. The amount of the light coupling into secondary fibers is measured through power meters, which connected with secondary fibers. The humidity level has a direct impact on the R.I., further change in R.I leads to produce a variation in the power coupling from primary fiber to secondary fiber. Where, to effectively change the R.I., the chemical coating is applied on the twisted fibers air gap.

#### Bend loss

The bend radius has a greater influence on varying the radiated light from illuminating fiber. There's numerical and practical proof that a smaller bend radius causes more radiation loss. In the illuminating fiber, bend loss is caused by macro-bending and cladding modes that have generated radiation. When it comes to macro-bending radiation modes, the basic cause of rays are refracted rays and tunnel rays^33^. The active fiber has losses due to the bend structure of the fiber, where the losses from the active fiber could be gained by the passive fiber by the coupling effect. For coupling, the passive fiber needs to be tightly bound with an active fiber. As the maximum light amount could be coupled into passive fiber through the side coupling method. The bending of the fiber produces the radiation field, which is led radiation field in the surrounding areas. Thus, the light in the secondary fiber is coupled by the formation of the radiation field by the illuminating fiber. While the secondary fiber is free from any light source.

Instead of tapering and side-polishing, twisted structure is used^29,30^. By twisting rather than placing two fibers parallel, the fibers are gripped better; at the same time, twisting improves coupling effect and stabilizes coupling coefficient. The coupling power is expressed as^31^.

1 $${{\text{P}}}_{{\text{c}}}=\sqrt{1+{\left(\frac{{\text{C}}}{\sqrt{{\text{nC}}}}\right)}^{2}}$$

where ${P}_{c}=$ coupled power, ${\text{n}}$= effective refractive index, and $C$ = coupling coefficient. Thus, the coupling can be varied if the refractive index is changed. After twisting, the usually refractive index is considered to be 1 due to air medium in our system. Now, as humidity rise which can change the refractive index. So, there is intensity variation due to change in refractive medium.

## Results and discussion

The present work discusses the results of the dual point relative humidity sensor. The sensor is designed from POF by using PMMA fiber. Previously, several researchers have applied the POF sensor for single point humidity measuring technique. The uniqueness of the present work is to measure the humidity at two different positions through dual point humidity sensor. The results of present dual point humidity sensor are discussed here to know the ability of proposed sensor toward humidity measuring. The present paper combines the results of dual point humidity sensor with and without chemical treated sensors. For chemical treated sensors agarose-gel is coated on twisted fibers to improve the sensitivity of the sensor. The proposed dual point humidity sensor of the present study measures the humidity variation based on the change in the R.I. The R.I of air is usually considered as 1. Where increasing humidity level increases the R.I. By varying R.I, the coupled power varies from one fiber to another fiber. Twisted fibers make up the basic structure of the sensor. The twisted fiber was bent up to a radius of ~ 10 mm. To easily bend the sensor up to the radius of 10 mm, the fiber should be a soft and flexible material. Mitsubishi SK-40 fiber has significant advantages, such as being able to be bend at a minimum bend radius with maximum bending loss. Due to the small cladding region and large core diameter, it can gain the loosed light by side coupling. These properties of POF made it most suitable to design the dual point humidity sensor. While, the Glass Optical Fiber (GOF) couplers must be specially treated when they’re made, compared to POF couplers^32^. To shield any possible interference from external light, GOF free ends need to be covered with a black jacket^33^. While the POF is free from special treatment for side light coupling.

### Without agarose-gel

**Figure 3:**
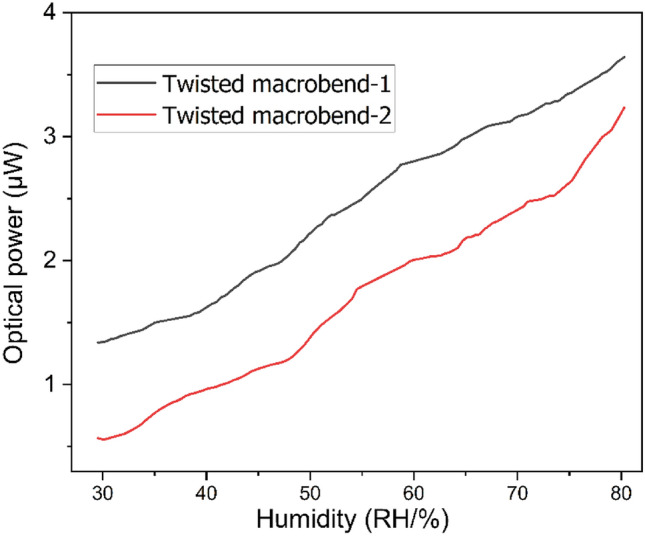
Dual point humidity sensor's response without coating of agarose-gel.

The humidity is the level of the water vapor contained into the air. The level of water vapor increase in the surrounding environment leads to an increase the humidity level. Figure 3 shows the response of the dual point relative humidity sensors. The dual point relative humidity sensors consist of the two sensors TMB-1 and TMB-2, with the measuring range of humidity from 30 to 80%. The humidity between 30 and 80% has a very linear response. Where humidity below 30% has a very low percentage of water vapor, which insignificantly changes the sensor response. Thus, the present work is considered the sensor for measuring the humidity from 30 to 80%. As humidity increases, the coupled intensity also increases because the more radiated loss from illuminating fiber is being coupled in passive fibers. Both sensor structures have active responses of optical power coupling concerning the variation of humidity level. Compared with TMB-2, TMB-1 has more sensitivity. In the sensor design structure, the TMB-1 has higher light intensity as compared to TMB-2. The difference of light intensity in both sensors is due to the passive fiber twisted with active light source fiber. The first bend loss occurs in TMB-1 and the second bend loss occurs in second band. However, in case of multiple bending, then optical power will gradually decrease.

### With agarose-gel

In sensors technology, sensitivity is a critical parameter that directly impacts the sensor's ability to detect and measure changes in physical parameters such as temperature, humidity, turbidity etc. When it comes to fiber optic sensors based on twisted fiber structure, sensitivity remains an important concern, and it influences the overall performance and usefulness of the sensor. A number of different techniques could be applied to increase the sensitivity of the sensor. Firstly, increasing the input LED power in a POF could improve the sensor's sensitivity. But it's important to consider the trade-offs and limitations associated with this approach. POF sensors operate based on the principle of light interaction with the sensing element, which can be affected by various factors, including the intensity of the light source (LED in this case). At very high-power levels, some nonlinear effects can occur in both the polymer material and the fiber itself, potentially leading to distortions in the sensor's response and reducing accuracy. Besides increasing the input LED power, coating the fiber with chemical substance is another possible solution to increase the sensor's sensitivity.

**Figure 4:**
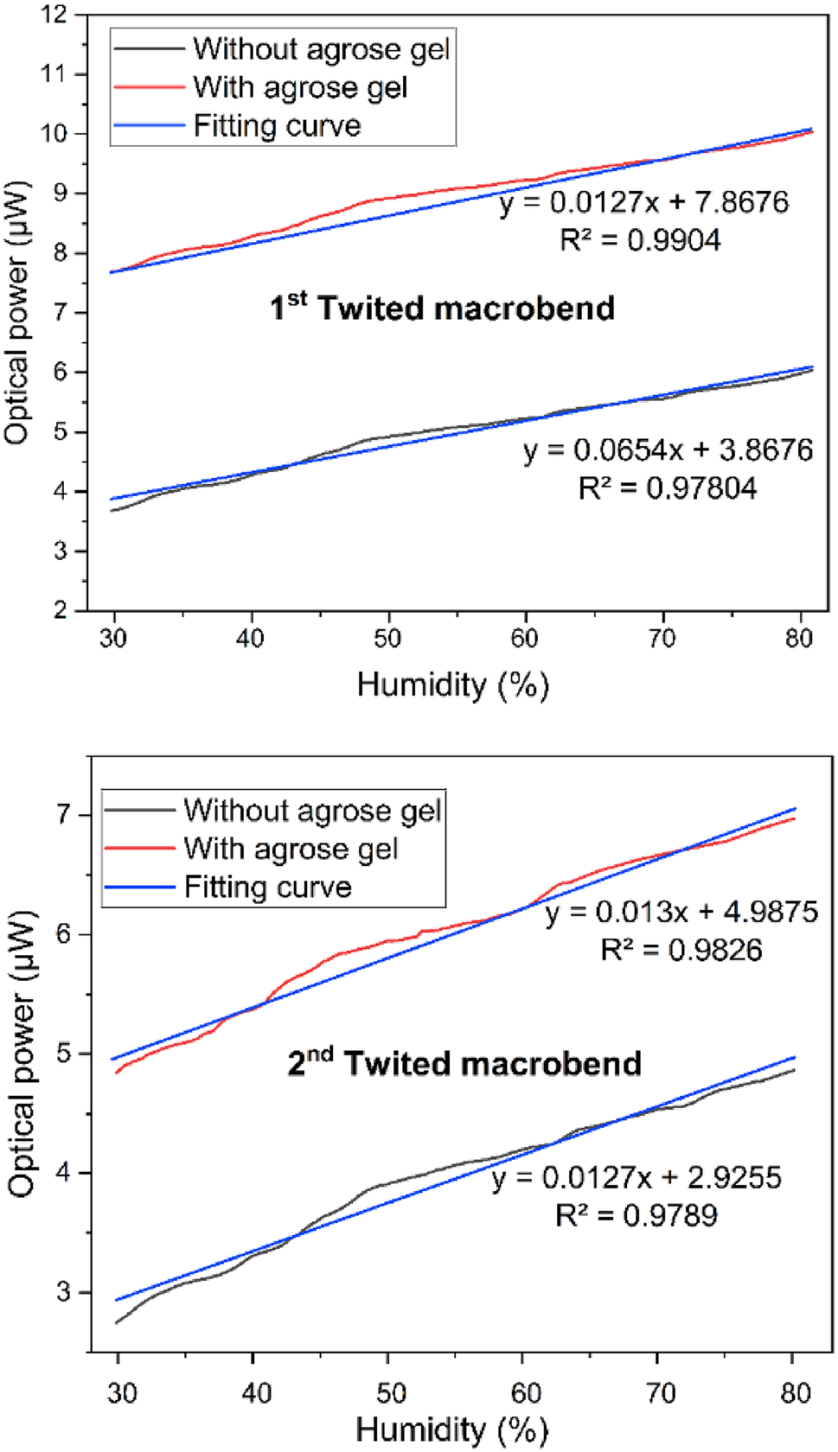
Comparison of humidity response between chemical treated and without chemical treated dual point sensor.

Figure 4 compares the humidity sensing of the dual point sensor between chemical treated (i.e. agarose-gel) sensor and without chemical treated sensor. Figure 4a,b represent the humidity sensing for TMB-1 and TMB-2. The dual point relative humidity sensor has a linear response of humidity between 30 and 80% range. Thus, to get a linear response with respect to change in humidity level, the preset work focuses on measuring the humidity in the 30% to 80% range. To compare the humidity sensing response, the both chemical treated sensors and without chemical treated sensors have variable sensitivity. Further, to compare the sensitivity between chemical treated sensor and without chemical treated dual point sensors, the chemical treated dual point sensors are found to be higher sensitive than without chemical treated dual point sensors.

For agarose-gel coated dual point sensors, when the humidity level rises, the agarose-gel absorbs the water vapors through a hydration process. The agarose-gel's hydrophilic property caused the interaction of water vapor with the gel matrix. The interaction of water vapor with the gel matrix leads to gel swell. The swelling of gel has increased the volume and size of the gel. Thus, this has increased the ability of twisted fiber to gain higher power through side-coupling. The increase in the ability of passive POF to gain a higher amount of power is due to the power lost from active fiber being reflected into agarose-gel. The swelling of the gel could also change the gel's physical property, affecting its performance during gel electrophoresis. To compare the sensitivity, the agarose-gel coated dual point sensors have better sensitivity than without agarose-coated dual point sensors. Table 1 presents the values of different parameters. The sensitivity of the agarose-coated probe sensors was about 680.8 nW/% and 763.9 nW/%. Moreover, the detection limit was about 1.37% and 1.98% for TBM-1 and TBM-2 agarose-gel coated. Overall, agarose-gel coated sensors are found to have significant advantages over without agarose-gel coated sensors. The advantages of agarose-gel coated sensors are due to increasing power coupling from primary fiber to secondary fiber.

**Table 1:** Performance of chemical treated and without chemical treated dual point humidity sensor.

	No	Performance parameters		
		Sensitivity (nW/%)	Std deviation (nW)	Limit of detection%
With coating	TMB-1	680.8	325.8	1.37
	TMB-2	763.9	372.7	1.98
Without coating	TMB-1	1052.1	306.7	3.43
	TMB-2	1274.4	302.6	4.21

### Repeatability response

**Figure 5:**
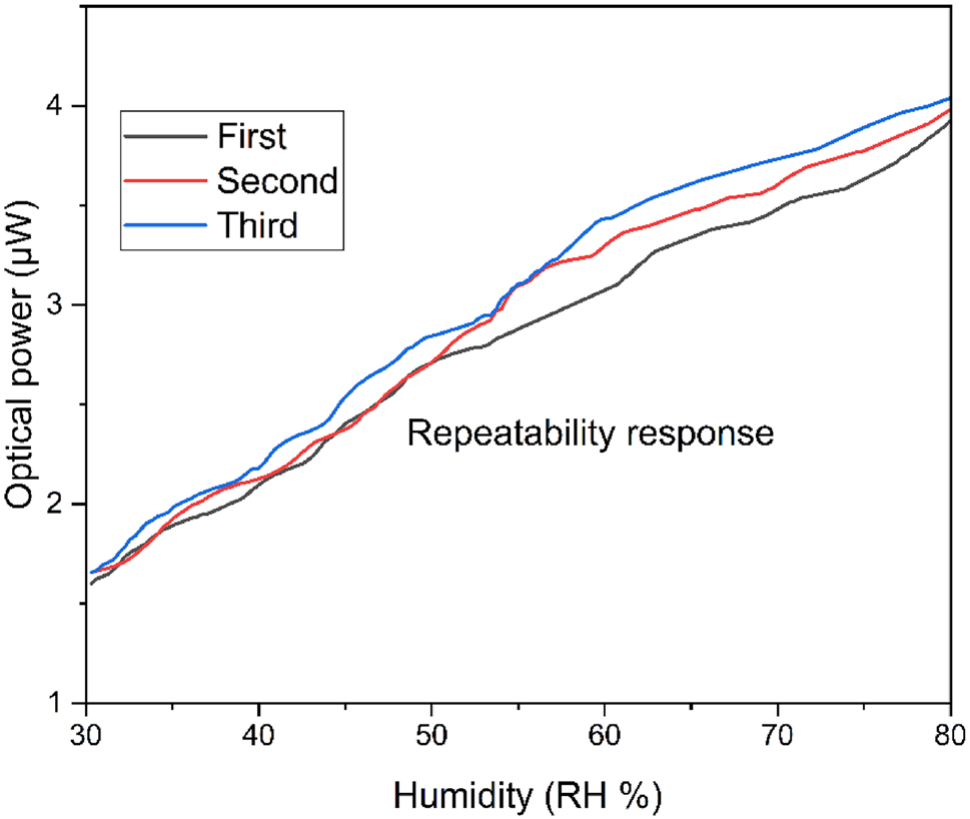
Chemical treated dual point humidity sensor repeatability response.

The sensor should be able to respond similar with repeatable experiments. The same repetition response of the sensor would indicate its importance. This highlights the sensor's reliability, and that the sensor consistently provides accurate measurements. Figure 5 shows the repeatability response of the chemical treated dual point humidity sensor. The three times repetition of the humidity measuring was carried out during the interval of 20 min. At all three repeatable times, the present chemical treated dual point humidity sensor has almost a similar response and there is very small variation. The variation is about ~ 10 nW which is quite small that could be ignored. The small variation in the repetitions humidity measurement response is due to the water vapor being slightly varies on the PMMA fiber and that effect on light coupling. However, the overall response of the sensor remains the same after multiple experiments to analyze repeatability response.

### Humidification and dehumidification response

**Figure 6:**
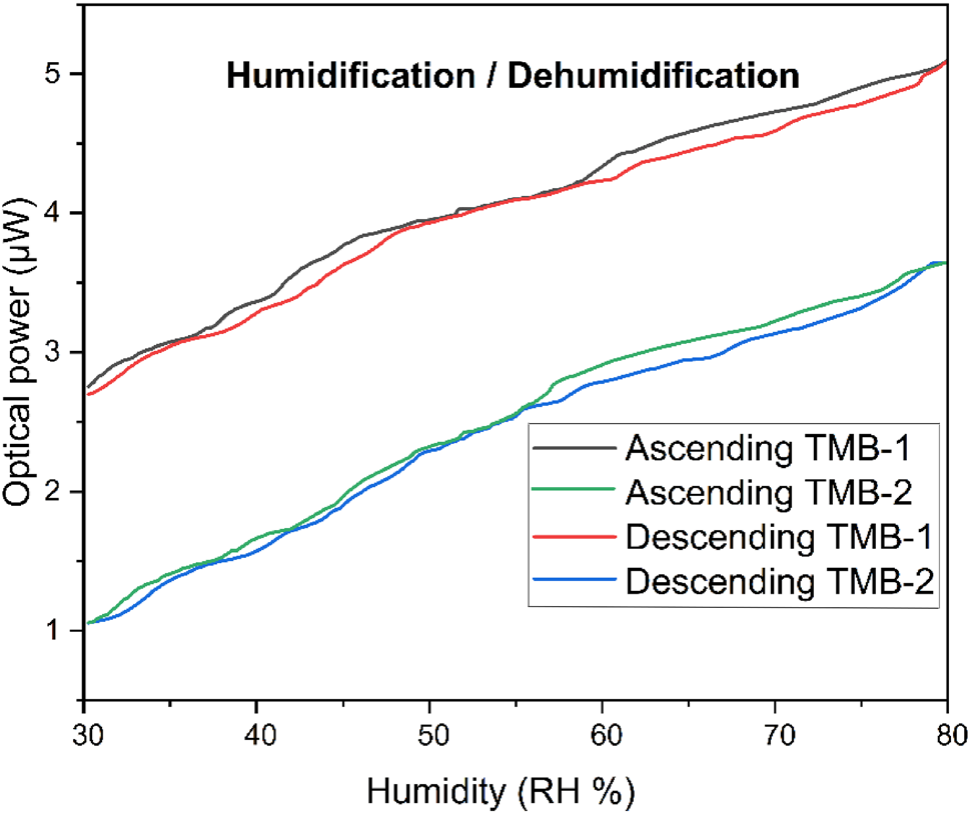
Dual point humidity sensor humidification & dehumidification response.

Humidity variation could be either in ascending or descending value. The sensor utilized for measuring humidity level should be able to respond linearly either an ascending or descending humidity level. As in the atmosphere due to rain and clouds the percentage of water vapor in the surrounding air significantly increase which leads to increase the humidity level. While, the strong sunshine has an oppositive nature, which reduces the water vapor percentage from air, which causes a reduction in the humidity level. Present study measures the dual point humidity sensor’s response regarding ascending and descending. Figure 6 shows the response of TBM-1 and TMB-2 to sense the humidity in ascending and descending values. The results obtained for both TMB-1 and TMB-2 indicate that humidity sensing has a very close response with ascending and descending values, which could be acceptable.

### Response time

**Figure 7:**
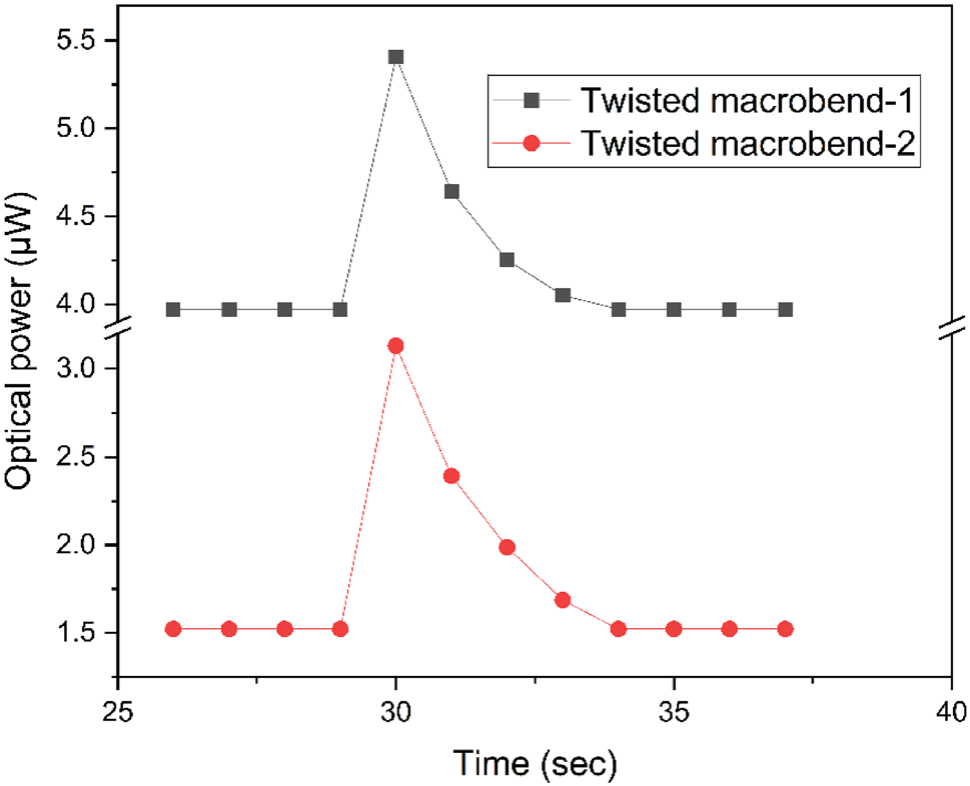
Dual point humidity sensor response and recovery time.

To design sensor for humidity measurement, it needs to be quickly response with respect to time. By exhaling a one-second breath into the probe, the humidity is changed to analyze the sensor's response time. Though this isn't the best technique for analyzing time response accurately, it provides a basic reference for time response analysis. Figure 7 shows the response time of both sensors TMB-1 and TMB-2. To measure the response time of dual point humidity sensor. The humidity in the chamber was set at 45% at the room temperature of 25 °C. The sensor’s response time is about 1 s, and recovery into the original position is about 4 s. When talking about the traditional sensors, the response time of the present dual point humidity designed sensor is faster.

### Dual point humidity sensor advantages

The present study has been proposed the dual point humidity sensor to sense the humidity at various point based on the single illuminating fiber. Further, to compare the single point base POF humidity sensor, the present dual point POF humidity sensor has the same humidity sensing performance. Considering the advantages, the dual point humidity sensor has significant advantages over the single point base POF humidity sensor. The advantages include the dual point humidity sensor measures the humidity at various positions based on the single illuminating fiber. Thus, this makes the system cheaper and simpler than single point base humidity sensor. Where, single point base humidity sensor requires a costly system for measuring humidity at various positions. Which requires the number of sensors integration. The present work’s dual point humidity sensor proposes the appropriate design of the sensors, which have high sensitivity, good repeatability, and significant response time.

## Conclusions

The present work is designed a dual point sensor from POF for the application of measuring humidity variation. The dual point sensor is able to measure the humidity variation at two different points through a single control system. The dual point humidity sensor of the present study combines the two twisted macro-bend structure sensors named as the TMB-1 and TMB-2. Both sensors measure the humidity independently through a single light input source. At initial, the dual point humidity sensor is used to measure the humidity through without chemical coating of agarose-gel on the sensor structure. The results indicate that the sensors have average sensitivity through without chemically treatment of sensors. Further, to improve sensor sensitivity, agarose-gel’s chemical coating is applied on both TMB-1 and TMB-2 structures. The chemical coating of agarose-gel was found to help the sensor to have close reversibility response in the dehumidification process. Further, for the repeated humidity measuring through chemical treated dual point humidity sensor is also found to be close response. The overall present proposal dual point humidity sensor design has a significant response to measure the humidity range from 30 to 80% with the appropriate response. To measure the sensitivity of the dual point humidity sensor with chemical coating of agarose-gel on TMB-1 and TMB-2 have a sensitivity of 680.8 nW/% and 763.9 nW/%. Further, the detection limit for both sensor structures measures about 1.37% and 1.98%. In future work, multiplexing of humidity measurements could be achieved.
