# Author Correction: Development of a dual point humidity sensor using POF based on twisted fiber structure

**DOI:** 10.1038/s41598-024-64544-0

**Published:** 2024-06-12

**Authors:** Sadam Hussian, Mujahid Mehdi, Abdul Ghaffar, Kun Lan, Yanjun Hu, Huan Lin, Mumtaz A. Qaisrani, Sikandar Ali, Jie Lin, Rehan Mehdi, Rui Ma

**Affiliations:** 1 Key Laboratory of Air-Driven Equipment Technology of Zhejiang Province, College of Mechanical Engineering, Quzhou University, Quzhou, 32400 Zhejiang China; 2 Faculty of Design, Aror University of Art Architecture Design & Heritage Sindh, Sukkur, 65200 Pakistan; 3 Taiyuan Institute of Technology, Taiyuan, China; 4 School of Mechanical & Materials Engineering, University College Dublin, Dublin, Ireland

Correction to: *Scientific Reports* 10.1038/s41598-024-59853-3, published online 10 May 2024

The original version of this Article contained an error in the name of author Kun Lan, which was incorrectly given as Lan kun.

The original Article has been corrected.

